# Probing Patient Messages Enhanced by Natural Language Processing: A Top-Down Message Corpus Analysis

**DOI:** 10.34133/2021/1504854

**Published:** 2021-05-18

**Authors:** George Mastorakos, Aditya Khurana, Ming Huang, Sunyang Fu, Ahmad P. Tafti, Jungwei Fan, Hongfang Liu

**Affiliations:** ^1^Mayo Clinic Alix School of Medicine, Mayo Clinic, Scottsdale, AZ, USA; ^2^Mayo Clinic, Department of Health Sciences Research, Rochester, MN, USA; ^3^Computer Science Department, University of Southern Maine, Portland, Maine, USA; ^4^Dubyak Center for Digital Science and Innovation, University of Southern Maine, Portland, Maine, USA

## Abstract

*Background*. Patients increasingly use asynchronous communication platforms to converse with care teams. Natural language processing (NLP) to classify content and automate triage of these messages has great potential to enhance clinical efficiency. We characterize the contents of a corpus of portal messages generated by patients using NLP methods. We aim to demonstrate descriptive analyses of patient text that can contribute to the development of future sophisticated NLP applications. *Methods*. We collected approximately 3,000 portal messages from the cardiology, dermatology, and gastroenterology departments at Mayo Clinic. After labeling these messages as either Active Symptom, Logistical, Prescription, or Update, we used NER (named entity recognition) to identify medical concepts based on the UMLS library. We hierarchically analyzed the distribution of these messages in terms of departments, message types, medical concepts, and keywords therewithin. *Results*. Active Symptom and Logistical content types comprised approximately 67% of the message cohort. The “Findings” medical concept had the largest number of keywords across all groupings of content types and departments. “Anatomical Sites” and “Disorders” keywords were more prevalent in Active Symptom messages, while “Drugs” keywords were most prevalent in Prescription messages. Logistical messages tended to have the lower proportions of “Anatomical Sites,”, “Disorders,”, “Drugs,”, and “Findings” keywords when compared to other message content types. *Conclusions*. This descriptive corpus analysis sheds light on the content and foci of portal messages. The insight into the content and differences among message themes can inform the development of more robust NLP models.

## 1. Introduction

Patient portals are secure online systems that enable patients to conveniently interact with their providers and access their medical records [1]. They have been gaining momentum in electronic health records (EHRs) in recent years due to the high priority of developing comprehensive health information technology and infrastructure. Early adopters of the patient portal system included Kaiser Permanente and the Veteran’s Affairs health systems [2]. Patient portals are starting to be seen as a necessary competitive edge for hospitals to improve patient experience via the ability to view notable labs, test results, and other personal health information pertinent to their care [2-4].

A popular feature of patient portals is “secure messaging,” a type of asynchronous communication between patients and providers between personal visits. Patients use this feature to ask clarifying questions, handle administrative elements, and even bring up new medical concerns. While this technology becomes more widely adopted, there is an inherent increase in the number of messages generated by patients across this platform. Institutions will need to have comprehensive strategies to handle the inflow of the messages and properly route these messages to the appropriate inboxes and recipients. Departments will also be faced with maintaining a high level of patient satisfaction metrics by attending to the messages within a timely manner, an additional responsibility on top of their clinic hours.

The use of artificial intelligence (AI) and natural language processing (NLP) to develop clinical support systems can aid in enhancing the impact of this technology in healthcare applications. These advanced statistical and machine learning techniques can facilitate content analysis by classifying patient-generated messages and extracting salient information from them. This approach has already been applied to other types of medical text, such as provider-generated input into the EHR. For example, one study used NLP for the identification of patients who experienced silent brain infarctions based on annotated neuroradiology reports [5]. The literature up to now has focused on provider-generated text because there is a more reliable standardization of medical vernacular amongst physicians [6].

Though there are many studies examining the logistics and interactions of patients with the secure messaging platform, there are few studies examining the content of the messages being sent by patients. A popular motif is to create models that classify messages [1, 7] and overarching concepts [8-10], but fail to provide a robust analysis of underlying keywords, imperative pieces of data for understanding the nature of the patient’s concern and thought process. For example, Shimada et al. determine message themes among patient-generated message threads at their Department of Veterans Affairs facilities [11]. However, this work analyzes “themes” in their content analysis without extracting specific keywords of phrases. Other patient-authored message analyses from the Mayo Clinic by North et al. have focused on the metadata of patient messages, such as the increased use rate of patient portals, which staff members respond to messages, and if response messages by staff are being sent after-hours [12]. More granular analyses of message content can better help clinicians understand patients’ concerns and needs. Furthermore, these analyses are integral to developing patient-centered informatics solutions.

In order to make these applications more robust for patient-authored messages, a much deeper understanding of the message content is necessary. Here, we evaluate the content of patient-generated portal messages (PPMs) in three typical departments with large message volumes through manual annotation of a selected corpus and extraction of key information from message content using NLP techniques. We performed a top-down analysis of message data in terms of departments, message content types, medical concepts, and keywords. We aimed to answer the following questions: (1) which message types, medical concepts, or keywords tend to be found more frequently in the message corpus? (2) What is the distribution relationship between message types, medical concepts, and/or keywords across three clinical departments?

Analyzing message content types enables us to compare the medical information provided in each type of message for better sorting and eventual triaging to the correct destination inbox; analyzing medical concepts and keywords helps us to better understand the valuable content patients mention within messages. In addition, an annotated corpus lays foundation for the development of an NLP pipeline for automatically classifying and triaging messages and extracting important content from them. This preparation can increase clinical efficiency and the quality of patient-centric care.

On a daily basis, clinicians interact with extractable and insight-rich data from multiple sources (if not all sources). If clinicians are aware of how informatic techniques can allow them to uncover trends and insights in this data, the likelihood of collaboration between clinical medicine and informatics to enhance patient care increases. Our goal is to demonstrate to a clinician audience a useful intersection of technology between informatics and NLP that can extract patient-related insights and identify potential applications from previously unstructured, patient-generated text.

## 2. Methods

### 2.1. Data Collection

Patient portal messages (PPMs) were extracted from the Unified Data Platform (UDP) of Mayo Clinic, Rochester, MN. With approval by Institutional Review Board (IRB# 18-009868), the 1000 most recent PPMs containing >80 characters from three clinical departments were extracted on Dec. 23, 2018: Cardiology (Oct. 16-Dec. 23, 2018), Gastroenterology (Nov. 27-Dec. 23, 2018), and Dermatology (Aug. 22-Dec. 23, 2018). We extracted PPMs from these three departments because they received the highest average yearly volumes of PPMs. We discarded six messages due to different written language, missing delimiters, and message duplication, resulting in 2994 portal messages for analysis.

### 2.2. Annotation Guideline Development and Corpus Annotation

In combination with domain knowledge, we followed a standard data abstraction and annotation process [5, 13]. We generated our annotation guide (see Supplemental Material (available here)) using details gathered from discussions with physicians, nurses, informaticists, and electronic medical assistants. Six medical students were recruited to label content types as annotation guidelines were continually developed. The initial training was provided by an experienced informatician (S.F.) to all annotators, covering overview of the annotation process, annotation instructions, and annotation guideline and tool. The annotation task was performed using a Multidocument Annotation Environment (MAE) [14] to label each message with one of four message content types: Active Symptoms (A), Prescriptions (P), Logistics (L), and Update/Other (U) as listed in Table 1. These content types were chosen based on discussions with clinical staff as aforementioned. Briefly, “Active Symptom” was assigned to messages that mention currently active symptoms; “Logistical” to messages that dealt with scheduling appointments, insurance questions, faxing documents, and other desk-staff-related tasks; “Prescription” to prescription-related messages; and “Update/Other,” a catch-all type, to messages of thanks and salutations or messages that did not fit into the A, P, or L types.

**Table 1:** Definition of message types.

Message types	Example identifiers	Message examples
Active symptom (A)	Pain, headache, hurts, pneumonia, rash	“I’ve been having real bad back pain.”
Prescription (P)	Prescription, medication, refill, pharmacy	“May I get a refill of my meds, please?”
Logistic (L)	Schedule, cancellation, record, insurance	“Can I reschedule my appointment?”
Update/other (U)	Non-medical, thanks, salutations	“Thank you doctor, you are amazing!”

If a message could be labeled with multiple labels, we instructed annotators to assign one label that reflects a majority of the message’s content. In order to achieve acceptable interannotator agreement (IAA) scores, we randomly sampled a set of small batches (i.e., 15, 35, and 50) of messages from our cohort for duplicate labeling among the annotators. After each round of labeling, an adjudication session was held to discuss discrepant labels and further standardize annotation guidelines. The remaining cohort was divided into three batches, the first of which included 500 messages. Of these 500 messages, 40% (200) were duplicates in order to detect IAA (F1 score = 0.7692).

The F1 score for IAA was calculated by using the harmonic mean of precision and recall in an averaged, pairwise fashion. For example, for two annotators, if we treat one as the subject (person 1) and temporarily treat the other annotator’s answers (person 2) as if they were a gold standard, one can calculate precision and recall for this scenario. From here, for >2 annotators, as in our study, pairwise F1 scores calculated in this fashion average to a total F1 score, with a higher score indicating higher interannotator reliability. More detailed description of equations and methodology used for this calculation can be found in the literature [15, 16].

### 2.3. Information Extraction

An open source named entity recognition (NER) software called MedTagger was used to extract keywords and corresponding medical concepts in messages. MedTagger uses a rule-based semantic lexicon [17, 18] to parse medical text. Like cTAKES or CLAMP, MedTagger is a generic information extraction framework and adopts a context detector developed by Chapman et al. with a sensitivity and specificity of 0.778 and 0.945, respectively [19]. MedTagger identifies words or phrases and assigns a Concept Unique Identifier (CUI) defined in the Unified Medical Language System (UMLS) [20, 21]. A CUI is associated with a normalized medical term. For example, the word “headache” or phrase “head pain” is identified from a portal message and assigned with a CUI “C0018681”. The CUI “C0018681” corresponds to a normalized medical term “headache”. The normalized medical terms (keywords) were further organized by medical concept (per MedTagger) via their semantic types (per UMLS). To clarify, the descending hierarchy of message data is as follows: (1) department (cardiology, gastroenterology, dermatology), (2) message content type (A/P/L/U), (3) medical concept of a keyword (i.e., FIND and ANAT), and (4) the keyword itself (e.g., “heart” and “lungs” are ANAT concepts).

### 2.4. Statistical Analysis

We used the identified keywords and associated medical concepts to determine any significant differences in their frequencies across departments and message content types. MedTagger further normalized the identified raw keywords (i.e., “both eyes” and “both of my eyes” were normalized to “both eyes”). First, the distribution of message types and medical concepts across the entire cohort and department subcohorts were measured. Second, the cumulative number of keywords for each medical concept was determined. Frequencies of keywords per medical concept and message type were computed. Third, keyword frequencies per message were calculated by dividing this cumulative keyword amount by the number of messages. Fourth, across the entire corpus, we identified the top 10 keywords within each concept. Finally, the top 30 most frequently found keywords within each department-specific message content type were identified. It was important to analyze the messages by department because each department subcohort could have its own unique set of keywords depending on the specialty.

## 3. Results

### 3.1. Distribution of Message Types

**Figure 1:**
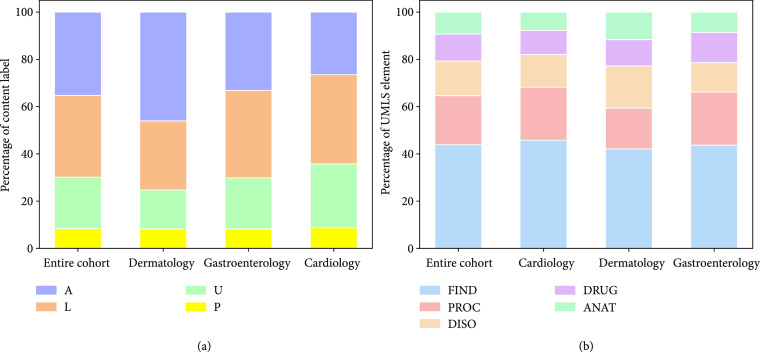
Distribution of message types (a) and medical concepts (b) across entire cohort and department subcohorts. (a) Distributions of Active Symptom (A), Logistics (L), Prescription (P), and Update/Other (U) messages are shown per the entire message corpus and per the cardiology, dermatology, and gastroenterology subcohorts. (b) Distributions of Findings (FIND), Procedures (PROC), Disorders (DISO), Drugs (DRUG), and Anatomical Sites (ANAT) are shown per the entire cohort and the three department subcohorts.

Figure 1(a) shows the distributions of message types—Active Symptom (A), Logistic (L), Prescription (P), and Update/Other (U)—in the entire cohort (2994 messages), the cardiology subcohort (997 messages), the dermatology subcohort (999 messages), and the gastroenterology subcohort (998 messages). The number of unique patient senders was 729, 750, and 746 for cardiology, gastroenterology, and dermatology, respectively. A majority (67%) of messages within the entire cohort were typed as “A” or “L”. The dermatology subcohort had a larger proportion of “A” messages when compared to the proportion within the entire cohort. Cardiology had a higher proportion of “U” messages. The “A” message type was found most frequently in the entire cohort as well as the dermatology subcohort. The “L” type was found most frequently in the cardiology and gastroenterology subcohorts. The “P” type comprised the smallest proportion of messages throughout the entire cohort and all three department subcohorts.

### 3.2. Distribution of Medical Concepts

Figure 1(b) shows that the distributions of medical concepts—Findings (FIND), Procedures (PROC), Disorders (DISO), Drugs (DRUG), and anatomical sites (ANAT)—were similar across the entire cohort and department subcohorts. FIND was the concept most frequently found in the entire cohort and within each department subcohort.

**Figure 2:**
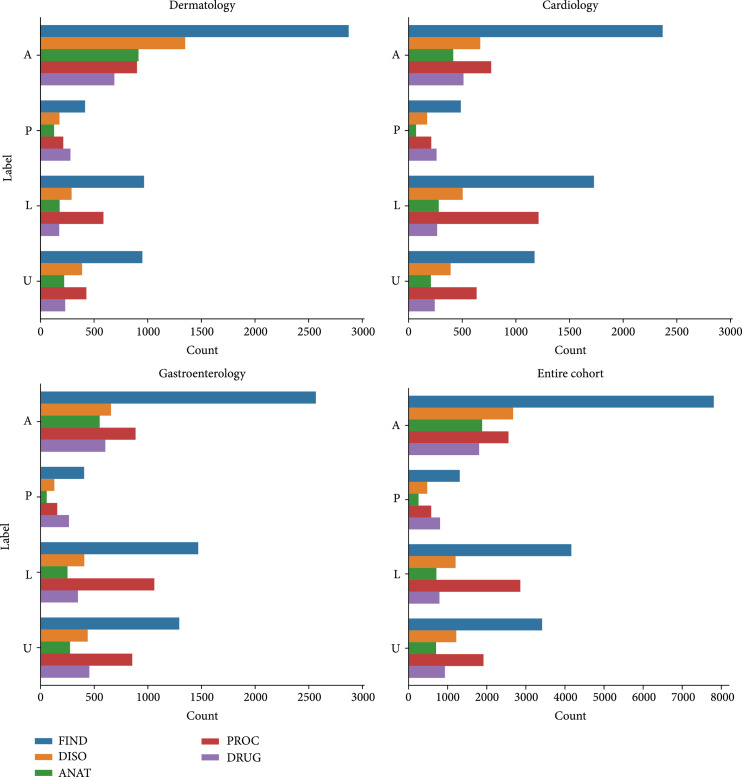
Frequencies of medical concepts among message types and departments. Frequencies of medical concepts across message types are shown for each of the three departments as well as the entire cohort.

Figure 2 visualizes the frequency of each medical concept based on message content types in the entire cohort and within department subcohorts. The FIND concept had the largest number of keywords across all groupings of content types and departments, having a total frequency of 16,695 instances in the entire cohort. The FIND concept includes a wide variety of keywords, including ones that pertain to laboratory or test results, attributes of the patient that may be related to their symptoms, and physiologic functions. ANAT and DRUG keywords were found less frequently overall at 3,549 and 4,327 instances, respectively. Each medical concept tended to have twice or thrice more keywords associated with it in messages that were labeled “Active Symptom” (A) versus “Logistical” (L) messages, even though the number of messages for each content type was similar.

In order to account for the varying number of messages in each content type, the total number of keywords within each message content type was normalized by the number of messages to provide the keyword count per message in Table 2. This shows the difference in proportion of MedTagger medical concepts among different content types. Messages labeled “Prescription” (P) tended to have the highest frequency count of DRUG keywords per message (3.21). ANAT and DISO keywords tended to be more prevalent in “A” messages (1.78 and 2.53 keywords/message, respectively). The highest proportion of FIND keywords per message tended to be in “A” messages within each department (8.97, 6.27, 7.75 keywords/message for cardiology, dermatology, and gastroenterology, respectively). “L” messages tended to have the lower proportions of ANAT (0.69), DISO (1.16), DRUG (0.76), and FIND (4.01) keywords per message when compared to other message content types.

**Table 2:** Frequencies of medical concept keywords per message in the entire cohort and the department subcohorts.

Cohort	Number of keywords per message				
	ANAT	DISO	DRUG	FIND	PROC
Entire (n=2994)	1.55	2.43	1.89	7.28	3.45
A (n=1055)	1.78	2.53	1.71	7.40	2.42
P (n=251)	1.01	1.90	3.21	5.22	2.31
L (n=1037)	0.69	1.16	0.76	4.01	2.76
U (n=651)	1.08	1.87	1.43	5.25	2.94

Cardiology (n=997)	0.98	1.74	1.29	5.77	2.84
A (n=264)	1.58	2.53	1.94	8.97	2.92
P (n=87)	0.81	1.99	3.00	5.60	2.43
L (n=376)	0.75	1.34	0.71	4.60	3.22
U (n=270)	0.77	1.45	0.90	4.35	2.35

Dermatology (n=999)	1.44	2.21	1.38	5.21	2.13
A (n=460)	1.99	2.93	1.50	6.27	1.96
P (n=82)	1.55	2.16	3.42	5.07	2.60
L (n=292)	0.61	0.99	0.60	3.31	2.01
U (n=165)	1.34	2.35	1.40	5.76	2.60

Gastroenterology (n=998)	1.14	1.63	1.67	5.74	2.96
A (n=331)	1.67	1.98	1.82	7.75	2.67
P (n=82)	0.70	1.56	3.22	4.95	1.89
L (n=369)	0.68	1.10	0.94	3.98	2.87
U (n=216)	1.27	2.03	2.10	5.98	3.95

### 3.3. Analysis of Message Keywords

**Figure 3:**
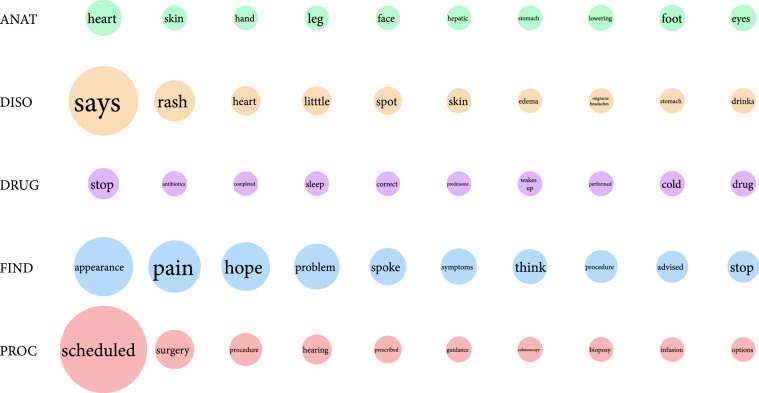
Unique keywords across all departments by medical concept. The top ten medical terms per medical concept are shown in each row, with the circles in each organized by decreasing frequency. The size of each circle is related to the frequency value for the keyword shown inside the circle, relative to “scheduled” as the largest circle.

Among the entire cohort, the top keywords from each medical concept category were “heart”, “says”, “stop”, “appearance”, and “scheduled”, respectively, as shown in the first row of circles in Figure 3. The keyword “scheduled” appeared a total of 546 times, indicated by the largest circle, whereas “foot” and “eyes,” the two smallest circles in the ANAT row, each appeared a total of 52 times. Many keywords were of the FIND concept as indicated by the relatively larger size of the circles.

**Figure 4:**
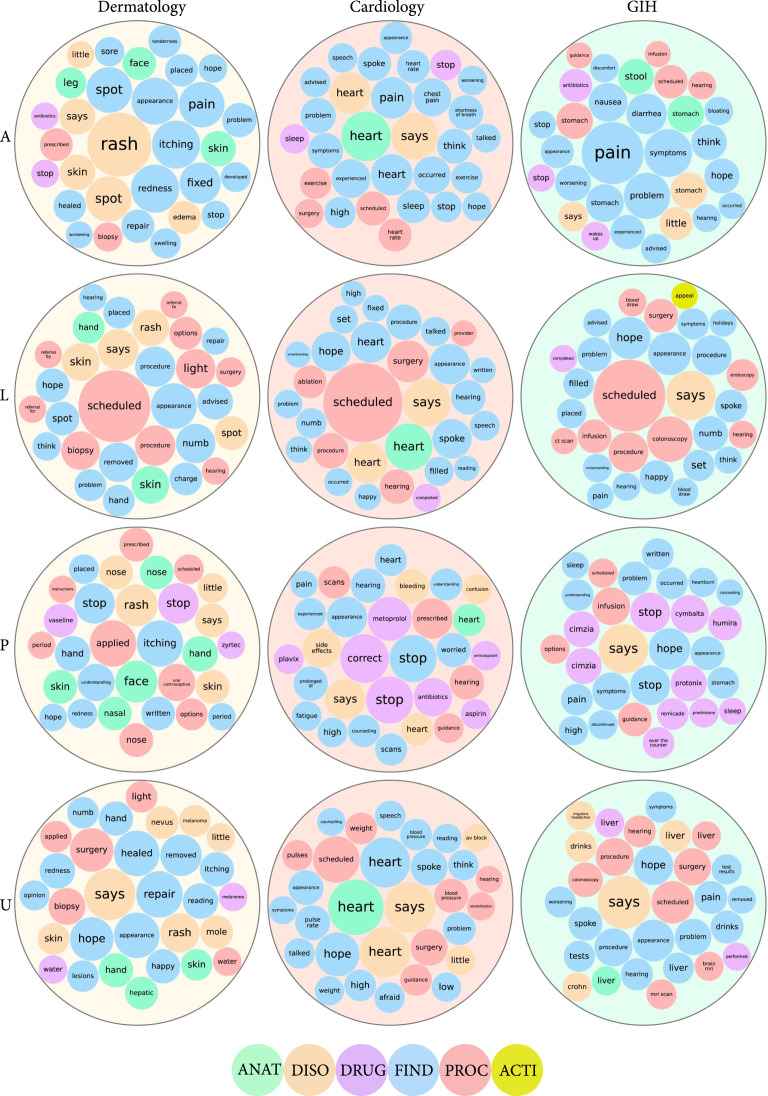
Keyword frequencies per department. The top 30 most frequently used keywords per department were identified by MedTagger. Each row represents the keywords from a particular message type (i.e., A/P/L/U) per department. Large circles represent department types and are colored light orange (dermatology), light red (cardiology), and light green (gastroenterology or GIH). Small circles are sized according to the keyword frequency value and colored according to the medical concept of the keyword (i.e., ANAT/FIND/DISO/etc.), using the same color scheme as shown in Table 1.

Figure 4 visualizes the keywords with the highest frequencies stratified by department and by message content type. The circle color indicates the type of concept to which the keyword belongs. This shows how the distributions of keywords and their medical concepts vary by the type of message in each department. Message content types differ in the distribution of keywords that appear within the message. For example, the word “scheduled” appears consistently within “L” messages, more so than other message types within the same department (each column). “A” messages tended to have high-frequency keywords that were specific to the biological systems of each specialty (e.g., “heart” for cardiology). Frequently used medications specific to each department appear in “P” messages. For example, “metoprolol” appears as a high-frequency keyword in cardiology, while “Cymbalta” is frequently mentioned in gastroenterology.

## 4. Discussion

Patient portal messages (PPMs) have become a common form of communication between patients and their caretakers. While they can increase convenience, PPMs have brought about unique challenges to healthcare communication structures. Messages can be long, complicated, contain multiple requests, and have a nondescript subject, which complicates downstream routing of PPMs. As machine learning (ML), deep learning (DL), natural language processing (NLP), and other AI techniques have been the focus of many technological paradigm shifts, healthcare remains a nascent landscape for applications.

### 4.1. Content Analysis for Insights in Clinical Practice

Automated annotation of PPMs is necessary to understand the stratified differences between departments (Figure 1). For example, cardiology was found to contain a higher proportion of “U” messages, which allows us to further evaluate what the patients authoring those messages are seeking from their care team. Comparing UMLS distributions within message content types provides technical characteristics that can aid in organizing. PROC keywords were found at about the same frequency within A and L messages. Perhaps this could be due to the higher likelihood that a patient writes about a procedure when talking about both a symptom they are having (i.e., “A CT scan showed…”) and an upcoming procedure that has been scheduled (i.e., “Can I schedule my lab test for Monday?”), whereas ANAT keywords, for example, would not tend to show up in “L” messages (Table 2). Additionally, as the FIND concept encompasses a broad range of medical terminology [22], it is expected that this medical concept is the most frequent (Table 2).

An analysis of the patient text corpus helps us understand patient behavior and optimize our clinical workflows accordingly. For example, if a care team’s goal is to reduce incoming message volume, high-frequency concepts can be used to utilize interventions that do not require the patient portal or redirect the patient away from sending a message and to an application that addresses their concern. Within the gastroenterology subcohort, “colonoscopy” was a keyword frequently found in messages labeled “L” (Figure 3). If the types of questions in these messages are regarding the preparation for a colonoscopy, new patient education material could be developed to answer patient questions so that they do not flood clinicians’ inboxes with simple, repetitive questions. There are instead other intrainstitutional educational outlets that can provide such information, similar to how Epic Systems MyChart mobile apps and the Wellpepper patient engagement community are used [23]. Continual message content analysis can guide intervention development and may be enough to significantly reduce the number of asynchronous communication requests. Additionally, keyword analysis can unveil critical, therapy-specific patient behavior outside the clinic such as extraclinical hormonal medication usage by breast cancer patients [22].

For the patient’s benefit, keyword analysis can elucidate the exact content of PPMs to update patient education materials, suggest resources upon sending a message, and other useful patient-facing tools. It is important to dissect message content in order to understand any identifiable trends in requests. Although the few studies examining PPMs have focused on superficial analysis of messages (i.e., grouping messages by “medication renewal,” “test issue,” or “custodial”) [8, 11], further probing of which exact keywords are surfacing within these messages is useful in informing which kind of tailored patient resources are necessary.

### 4.2. An Annotated Corpus for Automated Tools

Our annotated corpus demonstrates that PPMs can be categorized by message content to aid in developing automated tools. In our manuscript, we intended to analyze patient portal messages using well-tested NLP tools. Traditional NLP tools that remain widely used in clinical concept extraction work [24] could provide us a useful baseline distribution of message types, medical concepts, and keywords across the selected departments. These automated tools can enhance three different aspects of PPM data management: (1) message organization, triage, and routing mechanisms; (2) back-end organization; and (3) the patient portal experience for patients and providers. Making this data management possible, automated annotation helps build an algorithmic foundation off of which numerous robust artificial intelligence algorithms can be designed. If efforts to achieve automated manipulation of PPMs are to be implemented in a layered fashion, we argue that the foundation would comprise annotation and keyword analysis as highlighted in this work. Higher levels of development would involve segmentation [10], text summarization [25], message synopsis generation, sentiment analysis [26], EHR integration [27], and possibly healthcare venue recommendation. Additionally, baseline annotation methods are needed to further parse out multiple message content types that may be housed within a single PPM. One must seek out recommendations of matching datasets with appropriate artificial intelligence and machine learning techniques (e.g., semisupervised online learning, adaptive off-the-shelf classifiers, and neural feature augmentation) to manipulate said dataset [28].

### 4.3. Limitations

As with all digital patient message analysis, some unique limitations exist. Patients do not use the same medical lexicon with which healthcare providers communicate within clinical notes, thus adding complexity to information extraction. Large variability in the meaning of patient-generated text exists, which can skew the urgency of the patient’s concern. If two patients complain of “headache” in a PPM, yet one is referring to something reminiscent of temporal arteritis symptoms and the other a cluster headache scenario, both eliciting quite different levels of urgency, downstream digital management of this concern becomes complicated. Integrating urgency identification into an algorithmic pipeline becomes important to address this issue. This uncertainty makes the segmentation task much more difficult to accomplish from a rigorous research perspective. Regardless, the data in these messages are invaluable in understanding patient interactions with their clinical care teams.

Applying NLP techniques to patient-generated medical concerns adds additional challenges because patients’ colloquial references are not as specific as the standardized vernacular of medical providers. Because the UMLS dictionary was constructed using physician-generated documentation, we were required to review the initial output to ensure that incorrect normalizations of patient words were not being registered. For example, a common word that patients would use frequently was “ER,” typically mentioned in a story where they or a family member had a recent trip to the emergency room. MedTagger, based on the UMLS dictionary, would occasionally map this word onto “Estrogen” or “Estrogen receptor,” as “ER” from a provider’s lens can be used to describe whether a patient’s breast cancer is ER (estrogen receptor) positive. Thus, the current tools used to extract keywords and acronyms require specific training from a patient corpus such as the one we generated to ensure proper normalization of medical keywords. We realize that not every possible component/keyword of a PPM will be adequately recognized by MedTagger using the current UMLS paradigm. Misfits in relation to that paradigm is an ongoing conversation in the field of clinical NLP [29]. Our team is currently working to apply more complex deep-learning-based named entity recognition techniques to maximize extraction ability and quality.

Another limitation lies in the fact that not every term in the messages was assessed for extraction of keywords: the terms not covered by UMLS were not addressed in this study as we sought to analyze the patient message content in an ontological context that we were familiar with (i.e., UMLS). Studying patient-specific language characteristics was not the primary focus of this work and is left to future research.

### 4.4. MedTagger Specific Limitations

MedTagger is an information extraction framework that was developed to extract keywords and concepts from provider-authored notes, developing a semantic lexicon in the process. We applied MedTagger to extract concepts from patient-authored messages based on the UMLS dictionary lookup technique. Since this is a generic task, there is innately more noise and colloquial terminology, and the mapping of some words to normalized concepts was imprecise due to the variation in context information. Postprocessing was not done in this case as there was no systematic way to ensure that all questionable terms were removed.

Due to the concept extraction architecture of MedTagger, it was possible for a normalized term to appear in more than one medical concept. For example, the normalized keyword “heart” is tagged to both the ANAT and DISO concept categories. The ANAT-type “heart” came from raw text input such as “cardio,” “cardiac,” “coronary,” and “heart.” The DISO-type “heart” came from the raw text input “heart,” unexpectedly. A similar situation occurred for the normalized keyword “stop,” appearing both in DRUG and FIND. This phenomenon could be due to the concept mapping strategy utilized by MedTagger, which is derived from BioTagger-GM [30]. Briefly, this concept mapping strategy utilizes the context of the surrounding sentence to extract certain medical concepts. Therefore, depending on this context, the same normalized keyword could be mapped to different medical concepts via MedTagger. The data we present here include keywords and their corresponding medical concepts without surrounding sentence context. Our analysis did not examine the context that informed MedTagger extraction, which is warranted in future work. As discussed here, content extraction informed by context continues to be a difficult problem in NLP.

## 5. Conclusion

Automated processes that extract information from these messages and subsequently improve downstream uses of the data are necessary. We still have large strides to make before reaching a higher degree of automaticity, but with artificially intelligent systems becoming more integrated [31], it is only a matter of time before virtual assistants (VAs) process PPMs and their nested requests—automatically ordering scans, scheduling labs, and prescribing simple medication.

Now that an annotated corpus of PPMs has been arranged and is being built upon, we can continue to explore the utility of machine learning tools in the context of PPM routing. The annotated corpus of PPMs also serves as a foundation upon which other ML/NLP tools can be refined for future research. We have begun to test other NLP techniques that use deep learning model architectures (e.g., spaCy, NLTK, BERT, GPT, StanfordCoreNLP, and RNN- and CNN-based tag decoders). We aim to use these technologies to build robust, on-demand clinical plug-in applications. We anticipate further development and manipulation of the annotated PPM corpus of this work can lead us to future clinical insight and pertinent application generation.

## Data Availability

Unfortunately, due to the nuanced, HIPAA-protected patient health information used in this study (patient portal messages containing private, protected information), we cannot provide access to this content at this time.

## Supplementary Materials

Supplementary Materials Patient Free-Text Annotation Guideline.
