# Supplementary material for: Probing Patient Messages Enhanced by Natural Language Processing: A Top-Down Message Corpus Analysis
Source: Health Data Sci. 2021 May 18;2021:1504854. doi: 10.34133/2021/1504854 (PMC10877700; doi:10.34133/2021/1504854)
Supplement: Supplementary Materials — Patient Free-Text Annotation Guideline. [file 1504854.f1.pdf]

Patient Free-Text Annotation Guideline

Revision 1.2

January 9, 2019

1. Background

More and more patients and caretakers are using portal messaging systems to communicate. With these innovations, there is much more time that a physician, nurse, or healthcare worker has to spend sifting through messages to identify what a patient may need. Many of these messages tend to be quite long, requiring extensive review. As branches of artificial intelligence, such as natural language processing, are being used to streamline laborious processes brought about by these new messaging systems, we aim to summarize patient portal messages for simpler digestion and review. By annotating patient messages for different symptoms, and the location, severity, duration, etc. of those symptoms, we can create a training data set that is needed to train a machine learning algorithm to continue to recognize symptoms from new patient messages.

2. Annotation tool

The annotation tool for this project is Multi-document Annotation Environment (MAE), a Java-based natural language annotation software package. MAE is a non-web-based annotation tool. All annotation tasks were defined in a document type definition (DTD) file. Due to its lightweight feature, the software can be easily shared and updated across multiple sites without configuration and testing.

Download: https://github.com/keighrim/mae-annotation
Installation tutorial: https://vimeo.com/309909879
Passcode: mae_demo

3. Instructions

Please use this document for annotation to make sure we are on the same page. The majority of the information will be in the method section. Be sure to check the supplementary materials as well.

Training: Each annotator will be given an initial 1 h training session and ask to annotate 10 messages from the initial guideline development set of 30 and to then compare their annotations with the gold standard. Questions raised from the training exercise will be used to refine the baseline guideline.

Annotation Flow:
Guideline Development Phase: In one week, each annotator will annotate **100** patient messages (33 for each specialty). First batch will be 15 messages; second batch will be 35 messages; third batch will be 50 messages. After each batch, inter-annotator agreement (IAA) will be calculated, a consensus meeting will be organized, and guideline will be revised. The process continues until a high agreement is reached.

Annotation Phase: Over the next three to four weeks, weekly batches of **100** patient messages (33 for each specialty) will be annotated. After the weekly assignments were completed, we computed IAA, resolved disagreements, and clarified the guidelines. Proposed timeline starts from **Jan 17-Feb 12, 2019**.

Adjudication: The final gold standard annotations will be created by combining the individual experts' annotations followed by adjudication of the mismatches. The jointly annotated training set notes are added to the gold standard but excluded from the final IAA computation.

Time: Please **record the time** spending annotating each document. Take your time and try your best to annotate all concepts and properties. Fill out Google Form after each annotation session you perform, no matter how long: https://goo.gl/forms/xme64hOL8fHoyxcz1

Proposed Schedule:

Sun	Mon	Tues	Wed	Thurs	Fri	Sat
**Jan 6**	7	8	9	10	11<br>Perform Kappa measure this week	12
13	14<br>Submit kappa to NLP group	15	16	17	18	19
20	21	22	23	24	25	26
27	28	29	30	31	**Feb 1**	2
3	4	5	6	7	8	9
10<br>Submit 1st batch	11	12	13	14	15	16
17<br>Submit 2nd batch	18	19	20	21<br>George & Ditty Submit to NLP		

For reference:
https://www.i2b2.org/NLP/Relations/assets/Concept%20Annotation%20Guideline.pdf

4. Guideline

4.1 Concept mention

Patients frequently have multiple requests within their messages and seem to use "Non-Urgent Medical Question" as their catch-all category. It is important to understand what the patient is asking for in the message, and in order to do that, we must label the notes with their content type.

OVERALL LABEL: Message Type (4 labels)

1. **Active Symptom**
2. **Prescription Question**
3. **Logistical**
    a. appointment, test results, insurance
4. **Update/Other**
    a. Only use this if nothing else fits
    b. Make sure update DOES NOT have active symptom that patient needs addressed.

Please save files like this in a folder named "Completed_(name)", with the message types in order of importance: put primary message type as first letter (APLU vs. LAPU)
A - active symptom
P - prescription question
L - logistical
U - update/other

samplemessage1_APLU.xml (message encompasses all four types)
samplemessage2_A.xml (message encompasses only active symptom)
samplemessage3_AL.xml (message encompasses an active symptom and logistical component)

Save message type by what the patient is asking for. What is the patient concerned about, what would be the provider's action item (Dealing with active symptom, answering prescription question, attending to logistical details, receiving an update from the patient without active symptoms)?

a. If patient alludes to a symptom, but is really asking for an appointment to be scheduled ASAP -> label as Logistical (see example note below)

Example message:
*Karen I had my ct scheduled for 12/10. However, my **pneumonia** came back over weekend and missed it. I went back in hospital on Tuesday 12/11. I have some tests scheduled for this Friday. Could i get the colon ct set up for Friday? Thanks. Randy*

This message does indicate some sort of active symptom, but it does not seem to be the patient's primary concern. Their primary concern is coordinating the CT scan for an upcoming appointment, and so this primary message type would be labeled as LOGISTICAL.
